# The Cry1Ab Protein Has Minor Effects on the Arbuscular Mycorrhizal Fungal Communities after Five Seasons of Continuous Bt Maize Cultivation

**DOI:** 10.1371/journal.pone.0146041

**Published:** 2015-12-30

**Authors:** Huilan Zeng, Fengxiao Tan, Yinghua Shu, Yanyan Zhang, Yuanjiao Feng, Jianwu Wang

**Affiliations:** 1 Department of Ecology, College of Agriculture, South China Agricultural University, Guangzhou, 510642, China; 2 Key Laboratory of Agroecology and Rural Environment of Guangdong Regular Higher Education Institutions, South China Agricultural University, Guangzhou, 510642, China; 3 Key Laboratory of Agro-environment in Tropic, Ministry of Agriculture, South China Agricultural University, Guangzhou, 510642, China; Estación Experimental del Zaidín (CSIC), SPAIN

## Abstract

The cultivation of genetically modified plants (GMP) has raised concerns regarding the plants’ ecological safety. A greenhouse experiment was conducted to assess the impact of five seasons of continuous Bt (*Bacillus thuringiensis*) maize cultivation on the colonisation and community structure of the non-target organisms arbuscular mycorrhizal fungi (AMF) in the maize roots, bulk soils and rhizospheric soils using the terminal restriction fragment length polymorphism (T-RFLP) analysis of the 28S ribosomal DNA and sequencing methods. AMF colonisation was significantly higher in the two Bt maize lines that express Cry1Ab, 5422Bt1 (event Bt11) and 5422CBCL (MON810) than in the non-Bt isoline 5422. No significant differences were observed in the diversity of the AMF community between the roots, bulk soils and rhizospheric soils of the Bt and non-Bt maize cultivars. The AMF genus *Glomus* was dominant in most of the samples, as detected by DNA sequencing. A clustering analysis based on the DNA sequence data suggested that the sample types (i.e., the samples from the roots, bulk soils or rhizospheric soils) might have greater influence on the AMF community phylotypes than the maize cultivars. This study indicated that the Cry1Ab protein has minor effects on the AMF communities after five seasons of continuous Bt maize cultivation.

## Introduction

Genetically modified (GM) crops were first commercially introduced in 1996 and are now cultivated in at least 28 countries [1]. Insect resistance is one of the primary traits of GM crops that have been genetically engineered to express insecticidal toxins derived from the spore-forming soil bacterium, *Bacillus thuringiensis* (Bt). At present, Bt maize is the most widely grown Bt crop in the world, with an area of 6.0 million ha [1–3]. However, the rapid and widespread adoption of Bt maize has raised concerns about the impact of GM crop cultivation on non-target organisms in the soil environment, such as bacteria [4–6], fungi [7–10], protozoa [11–14], nematodes [15–18], and soil invertebrates [19–21]. These soil organisms play important roles in maintaining plant health and soil fertility through the decomposition of organic matter and nutrient mineralisation [22, 23].

Arbuscular mycorrhizal fungi (AMF) are ubiquitous soil microorganisms that can form symbioses with 70%–90% of land plant species [24, 25]. They provide nutritional benefits to plants in exchange for carbohydrates from the host plant. In addition, it is well-known that AMF play crucial roles in pathogen and stress tolerance [26, 27] and in the growth and productivity of plants [28]. AMF are sensitive to the mixture of available host-plant species, because they rely on particular host plants [29]; thus, they are important organisms to study when assessing the risks of GM crops [30].

A few studies have assessed the effects of GM crops (primarily Bt crops) on AMF and revealed that GM crops may have positive or negative effects on the structure and function of the AMF community. For example, Turrini et al. [9] observed that the root exudates of Bt maize (event Bt176) significantly reduced the presymbiotic hyphal growth of the AMF, *Glomus mosseae*, compared to the root exudates of another Bt hybrid (event Bt 11) and non-Bt maize. Castaldini et al. [6] revealed that Bt 11 and Bt 176 maize roots grown in soil for 8 and 10 weeks had significantly lower percentages of colonised root lengths than those of the wild-type maize. Similarly, lower rates of AMF colonisation were also observed in the roots of nine different lines of Bt maize than those in their corresponding non-Bt parental base hybrids, and the reductions were not related to the expression of a particular Bt protein [31]. However, Tan et al. [32] indicated higher percentages (though not significant) of AMF-colonized maize roots in 5422Bt1 (Bt 11) and 5422CBCL (MON810) than in the non-Bt conventional parental line 5422; an analysis of the molecular biological data (DGGE and DNA sequencing) suggested that the *Glomus* communities of the plant roots and rhizosphere soils are associated with different maize genotypes, because significantly different *Glomus* communities were observed between different non-Bt maize lines, between different Bt maize lines, and between Bt lines and their non-Bt isolines. The discrepancy of these studies may result from the differences in the maize cultivars, the expression levels of the Bt proteins, the age of the growing plants, the location of the plants, the species of AMF, agricultural management practices, etc.

The above studies examining the effects of Bt maize on AMF were primarily conducted during a single season or year of plant cultivation. However, it has been reported that the Bt proteins can maintain their activity for 180 to 234 days [4]. Does multi-season cultivation of Bt maize have any cumulative effect on AMF? In our previous study we conducted an experiment to assess the AMF colonisation and community diversity in the roots and soils of conventional maize planted in the area where Bt maize straws were returned after five continuous seasons of Bt maize cultivation in sympatry [33], and did not show a significant difference in AMF colonisation throughout the sampling period (seedling, large bell, or mature stages). In this study, we assessed the colonisation and structure of the AMF community in maize roots and bulk and rhizospheric soils during the fifth season of continuous Bt maize cultivation to provide further information about how AMF was affected by continuous Bt maize cultivation, because the evaluation of Bt crop biosafety is a dynamic and long-term process.

We hypothesised that continuous cultivation of Bt maize will affect the composition of the AMF communities and result in a decrease in the diversity of the AMF community. AMF colonisation was also examined. The diversity and richness of the AMF communities in the soils and maize roots were studied after five seasons of continuous cultivation using a terminal restriction fragment length polymorphism (T-RFLP) analysis and AM fungi-specific primers that targeted the nuclear 28S rRNA gene. We also used DNA sequencing of part of the nuclear 18S small subunit (SSU) rRNA, part of the 28S large subunit (LSU) rRNA, and internally transcribed spacers (ITS) of AMF to determine the abundance and the composition of the AMF communities. Our aim was to provide additional insights into the evaluation of the risks to AMF posed by multiple seasons of Bt maize cultivation.

## Experimental Section

### Study site and maize varieties

The experiments were performed at the Agricultural Experiment Station (23°08′N, 113°15′E) of South China Agricultural University, Guangzhou, China. The soil was a red clay loam, containing 34.85 g·kg^-1^ organic matter, 1.16 g·kg^-1^ total N, 1.28 g·kg^-1^ total P, and 19.42 g·kg^-1^ total K, 95.16 mg·kg^-1^ available N, 68.86 mg·kg^-1^ rapidly available phosphorus, and 196.37 mg·kg^-1^ rapidly available potassium, with a pH of 6.0. Two Bt maize varieties were used: 5422Bt1 (event Bt 11) and 5422CBCL (event MON 810). Both Bt varieties expressed the Cry1Ab protein. Their conventional (non-Bt) parent line, 5422, was the control in this study.

### Experimental design and sampling

Two Bt maize varieties, 5422Bt1and 5422CBCL, and their conventional (non-Bt) isoline 5422 were planted for five consecutive seasons following a randomised complete block design, as previously described by Tan et al. [32]. Four replicates of ten individuals were planted for each maize variety. The three maize lines were planted randomly in each block, with 0.75 m between rows and 0.30 m between plants. The first season of cultivation started in September 2009, and the subsequent seasons each began 6 months after the start of the previous season (the second and third seasons of Bt maize cultivation started in March and September of 2010, respectively, and the fourth and fifth seasons started in March and September of 2011, respectively). The plants were irrigated every 3 days and 10 g of Norway compound fertilizer (nitrogen:phosphorus:potassium = 1:1:1[15%:15%:15%]) was supplied to each individual at planting, the jointing stage, and the large bell stage during every growth season. The weeds were removed by hand pulling, and no pesticides were applied. When the plants reached the mature stage, the whole plants were removed from the experimental field, with the exception of the roots of the maize cultivated during the fifth season. The soil of every plot was turned over between seasons after the plants were removed.

Sampling was conducted when the maize had matured in the fifth season of continuous cultivation. The samples from three individuals were pooled into one sample. Before storage, the roots of the plants were gently shaken to release the bulk soils that did not adhere to the roots. The bulk soils were passed through a 2-mm sieve, mixed thoroughly, and stored at -80°C. When they reached physiological maturity in December 2011, the fifth season, the plants were gently dug up, and the soils attached to the roots were collected. The roots were rinsed with tap water and were divided into two parts for the subsequent analyses (AMF colonisation, extraction of nucleic acids, and analysis of the Cry1Ab protein). For the analysis of the AMF colonisation, the root tissues were fixed with a 1:1:1 solution of formaldehyde:acetic acid:50% alcohol (FAA) and stored at 4°C. The root tissues were stored at -80°C prior to extracting the nucleic acids and analyzing the Cry1Ab protein content. After removing the small clumps of soil that adhered to the roots, the roots were oscillated in 0.85% sodium chloride at 200 rpm for 20 min to release the rhizospheric soils, which were then collected by centrifugation at 4000 rpm for 5 min. The rhizospheric soils were stored at -80°C.

### Molecular determination of the AMF community structure and microscopic determination of AMF colonisation

The concentrations of the Cry1Ab protein in the soils and roots were determined using an ELISA (enzyme-linked immunosorbent assay) method. The AMF colonisation in the roots was calculated by the magnified gridline-intersects method, which used 30 roots from each sample. The soil DNA was extracted from 0.5 g of soil sample using the FastDNA Spin Kit (BIO 101 Systems, California, USA), according to the manufacturer’s protocol. The root DNA was extracted from 0.1g of fresh root sample using the cetyltrimethyl ammonium bromide (CTAB) method. T-RFLP was performed using successive PCR (the first round used the LR1/FLR2 primers, and the second round used the FLR3-FAM/FLR4-HEX primers), purification to a final concentration of 150ng/μL, restriction enzyme digestion (*Mbo*I and *Taq*I) with a final concentration of 30ng/μL, and the samples were then loaded onto an ABI capillary sequencer (Applied Biosystems, California, USA), with LIZ-500 as the size standard. The SSU-ITS-LSU fragments were amplified with the SSUmCf and LSUmBr primers, and cloned into *Escherichia coli* and sequenced. All of the above methods and primer sequences were described in detail in Zeng et al. [33] and in S1 Table.

### Data analysis

Gene Scanner Software v1.0 was first used to visually inspect the quality of the T-RFLP data. Nonmetric multidimensional scaling (NMDS) was used to assess the similarity of the fungal communities in the root and soil samples. AMF richness, Shannon’s index, Simpson’s index, and evenness were also evaluated (see Zeng et al. [33] for more details). One-way analysis of variance (ANOVA) was performed using SPSS version 13.0 (SPSS Inc., North Carolina, USA).

The sequences were obtained through automated DNA sequencing in both the forward and reverse directions. The AMF sequences were identified using the BLASTn program provided online by NCBI. A total of 432 sequences were obtained from the bulk soil, rhizospheric soil, and root samples. To evaluate whether the sampling effort was sufficient, a rarefaction analysis was conducted using Mothur software [34]. The sequences were also analyzed to detect the operational taxonomic units (OTUs) using Mothur software, with a 97% identity threshold. The most abundant sequence from each OTU was selected as a representative sequence, and the representative sequences were deposited in GenBank (http://www.ncbi.nlm.nih.gov/) under Accession Numbers KJ701442 to KJ701505. The sequence of each OTU was named according to the species, sample type, replicate number and a number produced during the cloning process. The diversity of 12 sequences from each sample was evaluated using the observed species metric based on the unique OTU of each sample produced by the Mothur software. Shannon’s index, Simpson’s index, and the evenness index were also calculated by one-way ANOVA using the same SPSS software.

To elucidate the phylogenetic relationships between the representative sequences and the treatments, a phylogenetic analysis was performed using the neighbour-joining method (with 1000 bootstrap replicates) implemented in MEGA 5.0 [35].

## Results

### Determination of the Cry1Ab protein content

There were no significant differences in the Cry1Ab protein content in the rhizospheric soils (F_2, 8_ = 8.73, *p* = 0.02) and bulk soils (F_2, 8_ = 3.00, *p* = 0.13) of the Bt and non-Bt maize (see Table 1, the Cry1Ab protein concentrations in the samples collected from the rhizospheric soils of 5422Bt1, 5422CBCL, and 5422 were 2.94, 5.05, and 0.52 ng·g^-1^, respectively, and they were much lower in the bulk soils). However, there were significant differences the in rhizospheric soil samples from the two Bt maize lines.

**Table 1:** Cry1Ab concentrations in the rhizospheric soils, bulk soils and roots of the Bt and non-Bt maize harvested at the fifth season.

	Cry1Ab concentration (ng/g)		
	Root	Rhizo	Bulk
**5422Bt1**	4743.96±430.5b	2.94±0.66ab	0.44±0.16a
**5422CBCL**	6142.97±177.12a	5.05±1.14a	0.10±0.04a
**5422**	1.26±0.23c	0.52±0.18b	0.30±0.03a

The values are presented as means ± SEs (n = 3). The same letter in each column indicates that there was no significant difference at the 5% level using Duncan’s multiple range test. Root = Roots, Bulk = Bulk soils, and Rhizo = Rhizospheric soils.

There were remarkable differences in the Cry1Ab protein content of the root samples (F_2, 8_ = 143.46, *p* < 0.01) from the Bt maize and non-Bt maize harvested after the fifth season of cultivation (see Table 1, the concentrations of Cry1Ab were 4743.96, 6142.97, and 1.26 ng·g^-1^ in the roots of 5422Bt1, 5422CBCL, and 5422, respectively).

### AMF colonisation in the roots

**Fig 1:**
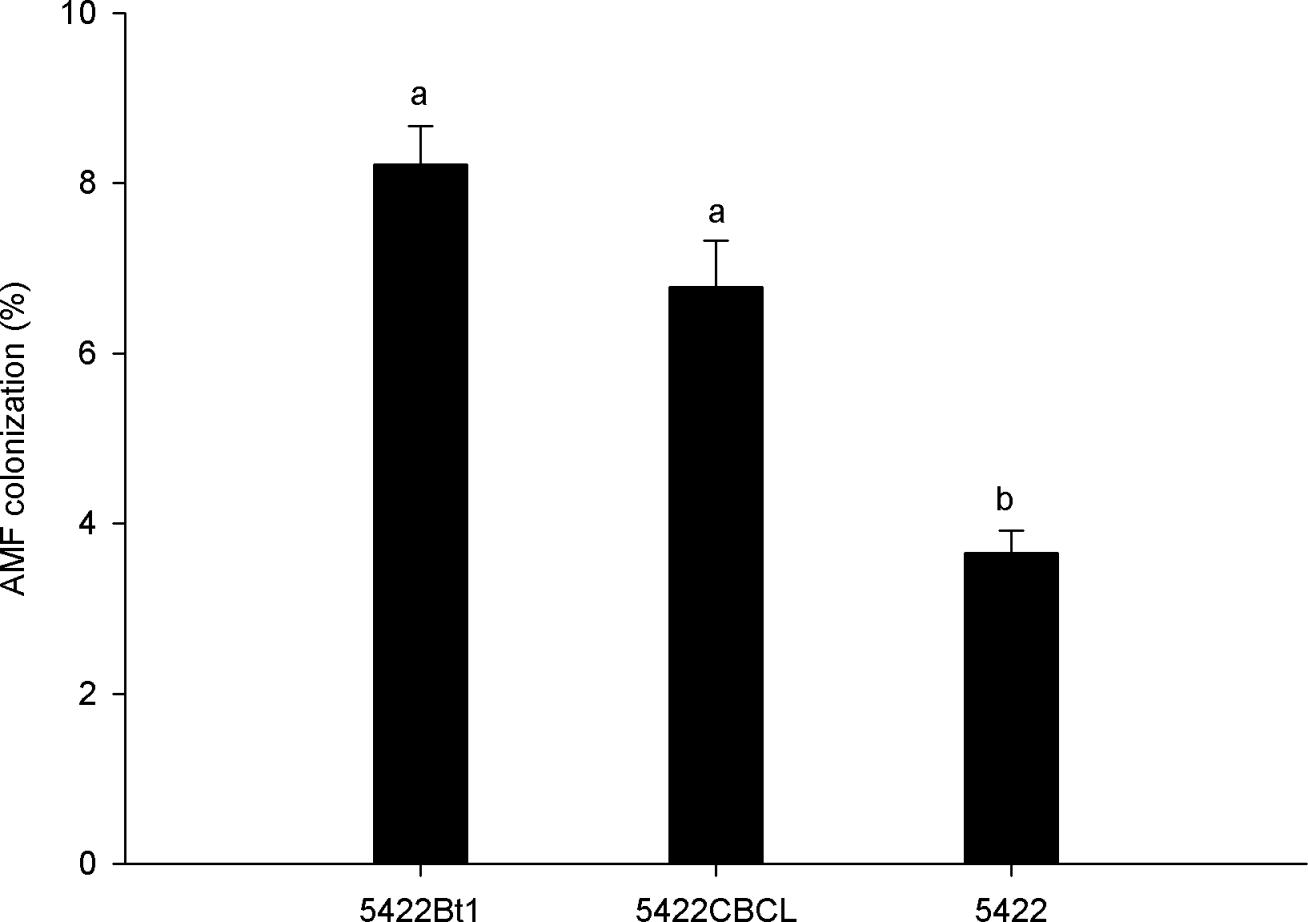
Percentage of AMF colonization in the roots of Bt and non-Bt maize harvested in the fifth season. The values are presented as means ± SEs (n = 3); the same letter in each column indicates that the differences were not significant at the 5% level using Duncan’s Multiple Range Test.

In general, the colonisation rates in the roots of all maize cultivars were not high (less than 10%, Fig 1). The two Bt maize lines (5422Bt1 and 5422CBCL) showed significantly higher colonisation rates than the non-Bt maize (5422) (F_2, 8_ = 28.74, *p* = 0.001). However, the difference between Bt maize lines (5422Bt1 and 5422CBCL) was not significant.

### AMF community diversity, as assessed by T-RFLP

A total of 25 unique terminal restriction fragment (T-RF) signals, which contributed to more than 1% of the total signal, were detected in the experiment. There were no significant differences in the AMF richness between the rhizospheric soil, bulk soil, or root samples of the Bt and non-Bt maize (Table 2).

**Table 2:** Richness based on the terminal restriction fragments (TRFs) in the roots and soils of the Bt and non-Bt maize harvested at the fifth season.

Variety	Restriction enzyme	Root	Rhizo	Bulk
**5422Bt1**	*Mbo*I	2.50±0.79a	3.38±0.55a	3.13±0.75a
**5422CBCL**		3.88±0.69a	3.88±1.39a	2.75±0.43a
**5422**		4.13±1.33a	4.00±0.74a	3.25±0.63a
**5422Bt1**	*Taq*I	5.63±2.44a	3.25±0.60a	3.13±0.75a
**5422CBCL**		3.75±0.43a	3.38±0.52a	2.75±0.43a
**5422**		2.75±0.48a	4.13±0.59a	3.25±0.63a

The values are presented as means ± SEs (n = 4). The same letter in each column indicates that the differences are not significant at the 5% level using Duncan’s Multiple Range Test. Root = root samples, rhizo = rhizospheric soil samples, and bulk = bulk soil samples.

Both Shannon’s and Simpson’s indexes showed that there were no significant differences in the diversity of the AMF communities in the roots, bulk soils, or rhizospheric soils of the Bt maize and non-Bt maize harvested in the fifth season of continuous maize cultivation (Table 3). The evenness index was only significantly different in the rhizospheric soil sample from the two Bt lines that had been digested with *Mbo*I.

**Table 3:** AM fungal diversity (Simpson’s, Shannon’s, and Evenness indexes) of the terminal restriction fragments (TRFs) in the roots and soils of the Bt and non-Bt maize harvested at the fifth season.

Sample types	Variety	Simpson’s index		Shannon’s index		Evenness index	
		*Mbo*I	*Taq*I	*Mbo*I	*Taq*I	*Mbo*I	*Taq*I
**Root**	5422Bt1	0.61±0.05a	0.55±0.04a	1.06±0.17a	0.92±0.14a	0.73±0.15a	0.54±0.23a
	5422CBCL	0.67±0.05a	0.70±0.03a	1.36±0.21a	1.40±0.10a	0.53±0.04a	0.56±0.04a
	5422	0.63±0.09a	0.62±0.05a	1.26±0.29a	1.10±0.13a	0.57±0.12a	0.60±0.13a
**Rhizo**	5422Bt1	0.63±0.05a	0.60±0.05a	1.19±0.16a	1.06±0.18a	**0.52±0.07b**	0.49±0.07a
	5422CBCL	0.71±0.09a	0.63±0.03a	1.52±0.35a	1.23±0.14a	**0.78±0.09a**	0.53±0.06a
	5422	0.7±0.06a	0.60±0.04a	1.42±0.22a	1.05±0.14a	**0.57±0.07ab**	0.59±0.14a
**Bulk**	5422Bt1	0.64±0.07a	0.61±0.06a	1.2±0.23a	1.11±0.21a	0.59±0.05a	0.49±0.07a
	5422CBCL	0.54±0.02a	0.56±0.04a	0.89±0.06a	0.93±0.12a	0.47±0.06a	0.43±0.05a
	5422	0.59±0.06a	0.56±0.03a	1.04±0.18a	0.95±0.11a	0.46±0.04a	0.44±0.04a

The values are presented as means ± SEs (n = 4). The same letter in each column indicates that the differences are not significant at the 5% level using Duncan’s Multiple Range Test. Root = root samples, rhizo = rhizospheric soil samples, and bulk = bulk soil samples.

**Fig 2:**
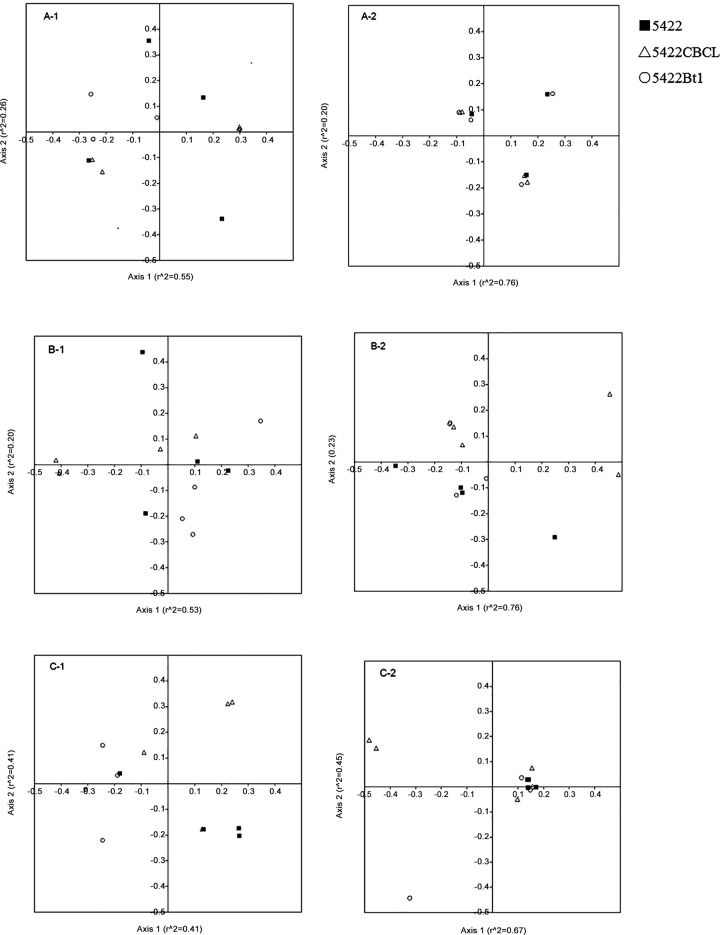
Nonmetric multidimensional scaling (NMDS) ordination plots of the AM fungal community structure, which is based on the TRFs for each enzyme, in the roots, rhizospheric soils, and bulk soils harvested at the fifth season. The filled square represents 5422, the circle represents 5422Bt1, and the triangle represents 5422CBCL. The key indicated the formula: sample type-restriction enzyme. A = bulk soils; B = rhizospheric soil; C = roots; 1 = *MboI*; and 2 = *TaqI*. The 95% confidence interval ellipses were not shown because the sample dot is too concentrated to distinguish if they were included (see S1 Fig to view the 95% confidence interval ellipses).

We also compared Bt and non-Bt maize community structure using non-metric multi-dimensional scaling (NMDS; Fig 2). The NMDS 95% confidence interval ellipses indicated there was no significant difference between the Bt and non-Bt maize (S1 Fig). The restriction-digested plots of the Bt and non-Bt maize NMDS were very similar, indicating that the distributions of the Bt and non-Bt maize were homogenous or overlapped. The result showed that after multiple seasons of cultivation, the AM fungal community structure in the Bt maize and non-Bt maize was not significantly different.

### AMF community diversity, as assessed by sequencing

**Fig 3:**
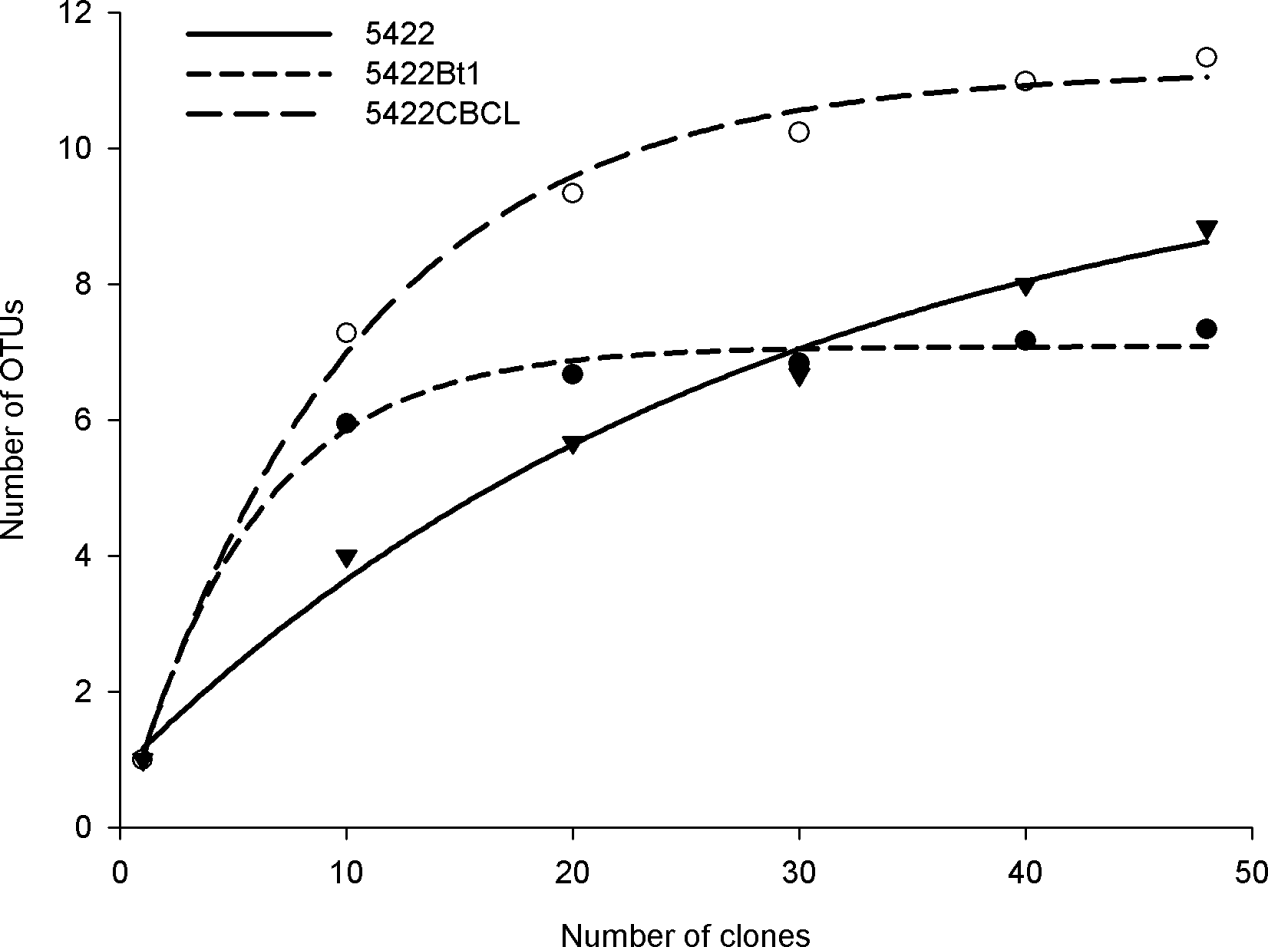
Sample-based rarefaction curve of the AM fungal community of the Bt and non-Bt maize harvested at the fifth season. The values are the average of the OTUs estimated in the roots, rhizospheric and bulk soils of each variety. A re-sampling analysis with replacement was performed using 1000 replicates in Mothur.

The length of the SSU-ITS-LSU fragment of the first PCR was approximately 1800 bp, while that from the second PCR was approximately 1500 bp. A total of 432 sequences were obtained from 36 samples (12 clones of each sample), which produced 64 OTUs with 97% similarity (29 generated from rhizospheric soils, 10 generated from bulk soils, and 25 generated from the root samples). The sample-based rarefaction curves had an asymptotic profile, indicating that the richness was stable in relation to the sampling effort of the study (Fig 3). The sample size of the selected clones in our experiment was large enough to reflect the putative differences in the community structure.

**Fig 4:**
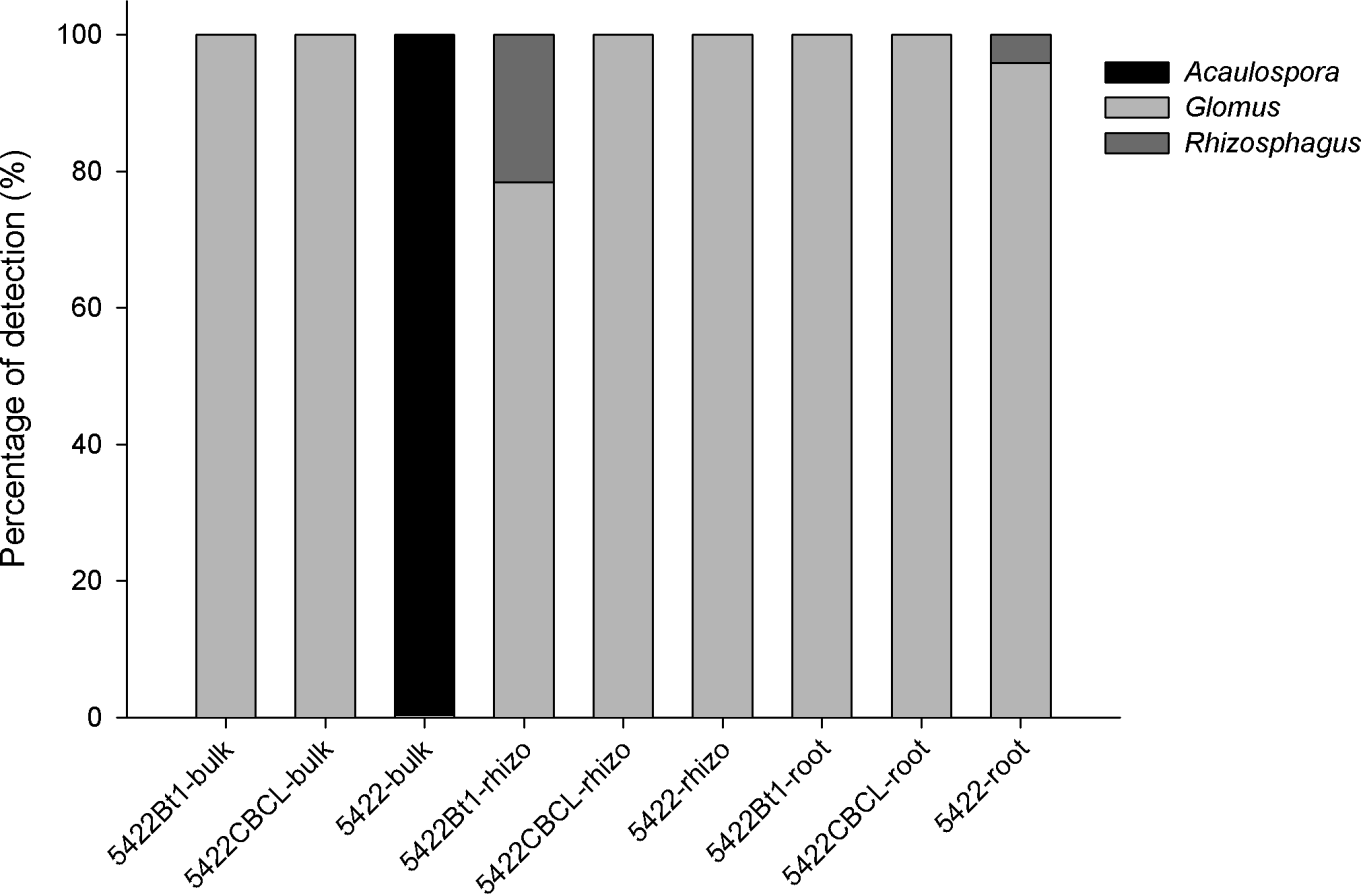
Percentage of the detected genera assigned to the AMF in the roots, bulk soils, and rhizospheric soils in the Bt and non-Bt maize harvested at the fifth season. The horizontal axis is labelled according to the following formula: maize variety–sample type (bulk = bulk soils, rhizo = rhizospheric soils, and root = roots).

The 432 sequences were blasted with the least similarity to produce a cluster around one species from each sample. The diversity indexes (Simpson’s, Shannon’s, and evenness indexes) calculated from the OTUs from each sample were not significantly different between the Bt and non-Bt maize (Table 4). The percentage of sequences belonging to each genus was calculated based on the total number of genera detected in each sample (see S2 Table for their phylogenic affiliations). Three genera, including *Acaulospora*, *Glomus* and *Rhizophagus*, were detected. *Glomus* was dominant in all samples, with the exception of the bulk soil of 5422, but the compositions and proportions varied (Fig 4). *Acaulospora* was only detected in the bulk soils adhering to the 5422 maize, while *Rhizophagus* was detected in the rhizospheric soil of 5422Bt1 and the roots of 5422.

**Table 4:** Simpson’s, Shannon’s and Evenness indexes based on the sequencing of the Bt and non-Bt maize harvested at the fifth season.

Sample types	Variety	Simpson’s index	Shannon’s index	Evenness index
**Root**	5422Bt1	0.72±0.02a	1.43±0.07a	0.85±0.05a
	5422CBCL	0.60±0.08a	1.06±0.25a	0.92±0.03a
	5422	0.67±0.07a	1.32±0.27a	0.85±0.06a
**Rhizo**	5422Bt1	0.52±0.05a	0.87±0.13a	0.85±0.06a
	5422CBCL	0.59±0.10a	1.02±0.27a	0.96±0.03a
	5422	0.42±0.05a	0.64±0.06a	0.87±0.08a
**Bulk**	5422Bt1	0.45±0.15a	0.77±0.26a	0.88±0.04a
	5422CBCL	0.41±0.12a	0.71±0.22a	0.78±0.05a
	5422	0.30±0.07a	0.47±0.08a	0.81±0.07a

The values are presented as means ± SEs (n = 4). The same letter in a column indicates that the differences are not significant at the 5% level using Duncan’s Multiple Range Test. Rhizo = rhizospheric soil; bulk = bulk soil.

Variety: 5422Bt1, 5422CBCL, 5422 maize.

Sample types: roots, bulk soils and rhizospheric soils.

**Fig 5:**
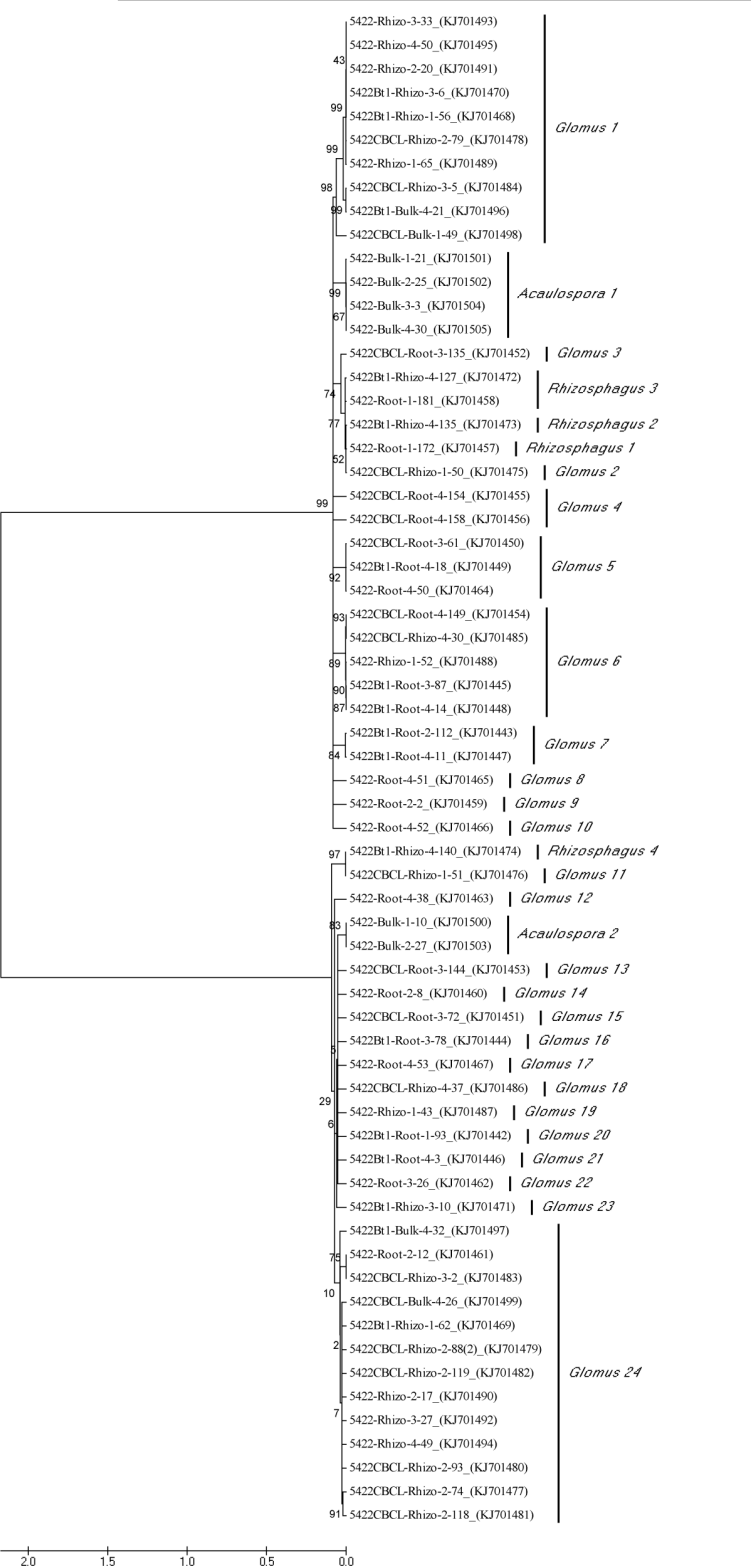
Neighbour-joining (NJ) phylogenetic tree (linearized tree) showing the relationships between the arbuscular mycorrhizal fungal phylotypes from the Bt and non-Bt maize that were inferred from the partial nuclear ribosomal DNA sequences of the 18S small subunit, internal transcribed spacer 1, the 5.8S ribosomal subunit, internal transcribed spacer 2, and the 28S ribosomal subunit. The bootstrap values above the branches are from the NJ analysis (1000 replicates). The *scale bars* indicate the average number of nucleotide substitutions per site. The sequence was also named according to the convention “maize variety-sample type-replicate number-the number generated in the cloning process (accession number)”.

The 64 identified OTUs were grouped into 30 discrete phylotypes (24 *Glomus* phylotypes, two *Acaulospora* phylotypes, and four *Rhizophagus* phylotypes) (Fig 5). No obvious clusters for the three varieties (5422, 5422CBCL and 5422Bt1) were revealed. The groups from most of the sequences generated from each sample type (roots, bulk soils or rhizospheric soils) were identified in the clustered tree. The bulk soil samples displayed two main phylotypes, *Acaulospora* 1 and *Acaulospora* 2. The maize roots were mainly colonised by 17 phylotypes (*Glomus* 3, *Rhizophagus* 1, *Glomus* 4, *Glomus* 5, *Glomus* 7, *Glomus* 8, *Glomus* 9, *Glomus* 10, *Glomus* 12, *Glomus* 13, *Glomus* 14, *Glomus* 15, *Glomus* 16, *Glomus* 17, *Glomus* 20, *Glomus* 21, and *Glomus* 22). Nine main phylotypes (*Glomus* 1, *Glomus* 2, *Rhizophagus* 2, *Rhizophagus* 4, *Glomus* 11, *Glomus* 18, *Glomus* 19, *Glomus* 23, and *Glomus* 24) were detected in the rhizospheric soils. Of these phylotypes, *Glomus* 5 was found in the roots of all three maize varieties.

## Discussion

### The accumulation of Bt proteins

The soils used in this study were collected from the garden of the Agricultural Experiment Station of South China Agricultural University, and thus, they are similar to natural soils. These soils may contain *Bacillus thuringiensis*, which can be detected using an ELISA kit. This was the reason why the Bt proteins were detected in the soils associated with the non-Bt maize 5422.

The significant differences in the concentration of the Cry1Ab protein between the roots from the two Bt maize indicated that the expression of the Cry1Ab protein varies in the two cultivars (5422Bt1 [Bt11] and 5422CBCL [MON810]) with different transformation events. The Bt protein that was released from the roots of the Bt maize may have been maintained in the soils because of the persistence and activity of Bt protein [4, 36–39]. However, the much lower Bt protein content in the rhizospheric soils and bulk soils than in the harvested roots suggests that there was no apparent accumulation of the Cry1Ab protein that had been released from 5422Bt1 and 5422CBCL in the soils. Another reason may be the difficulty of extracting the Cry1Ab protein bound to soil clays and humic acid using the PBST method, which results in the detection of lower concentrations of this protein in soils [36–38].

### AMF colonisation

The expression level of the Bt proteins in the roots of transgenic crops has been considered to be the primary influence on AMF colonisation [39]. Therefore, the significant differences in the concentrations of the Cry1Ab protein in the roots of the Bt and non-Bt maize could affect the maize-AMF symbioses. We found higher colonisation rates in the two Bt maize lines than in the non-Bt maize, indicating that continuous Bt maize cultivation had no adverse effect on AMF colonisation. The result was consistent with the study of Tan et al. [32] on the impact of Bt (5422Bt1 and 5422CBCL) and non-Bt maize (5422 and 5422wx) on AMF colonisation at four sampling times (36, 50, 64, and 78 days after sowing). In the rhizospheric soils, the Bt proteins may act as organic matter that fosters AMF colonisation and not as a toxin [4]. De Vaufleury et al. [39] reported that AMF were not sensitive to the expression of the Bt proteins in the roots of MON810 maize in a microcosm experiment. A similar result was also revealed in our previous study of the AMF colonisation in the roots of conventional maize planted in the area where Bt maize straws were returned after five continuous seasons of Bt maize cultivation in sympatry [33].

The AMF colonisation levels in this study were low (less than 10%) compared to those observed in a previous study by Tan et al. [32], which was conducted at the same location. The continuous turnover of the soil and farming practices for each season of maize cultivation might disturb the underground network of the AMF hyphae, resulting in the failure of the AMF to form hyphae bridges [40–42].

Our results were inconsistent with those reported by Cheeke et al. [31], who found lower root colonisation rates of Bt maize than those of their non-Bt parental lines. This discrepancy may result from the different soil types used in the experiments (50% local agricultural soil, 25% sterile sand and 25% sterile soil-less potting media in the study by Cheeke et al. *vs*. garden soil in the present study), the maize varieties examined (BC0805[Bt11] *vs*. 5422Bt1[Bt11], DKC50-20 [Mon 810] *vs*. 5422CBCL [Mon 810]), farming practices, and sampling times (60 d in the study by Cheeke et al. *vs*. maturity at approximately 90 d in the present study).

### AMF community composition and structure

The assessment of the AMF community using T-RFLP and sequencing has been widely employed in previous studies [43–45], but only a few primers can be used to monitor the entire AMF field community by sequencing the rDNA regions. The primers used in our study could amplify the rDNA fragments from the AMF in natural soils and are of sufficient length to facilitate our understanding of the risks posed by GM crop cultivation [46]. The amplified fragments included both conserved and variable regions, thereby providing information for AMF species classification.

In this experiment, we found 25 unique T-RF signals. We found fewer unique T-RF signals than Verbruggen et al. [47], who tested the potential effects of Bt maize on AMF communities using the same T-RFLP primers. The difference between these studies in the number of identified T-RF signals may be due to the differences in the clustering threshold of the peak area in each sample (1% clustering threshold of all peak areas *vs*. 0.5% clustering threshold of all peak areas by Verbruggen et al. [47]), the location of the experiments, or the maize varieties used. Other factors have also been reported that may influence the AMF richness, such as the fertilizer and farming practises. Stürmer and Siqueira [48] suggested that the mean species diversity and relative spore abundance are influenced by land use. Similarly, Oehl et al. [49] showed that a decrease in AMF species richness was related to more intensive cultivation. van der Gast et al. [50] demonstrated that the spatial scale of AMF diversity is also affected by farming practices.

In the present study, the insignificant differences in the richness and the diversity (Shannon’s index, Simpson’s index and evenness) of the AMF communities of the roots and soils between the Bt and non-Bt maize and the insignificant differences among the three maize cultivars revealed by the NMDS analysis suggest that there were no consistent or significant adverse effects of five continuous seasons of Bt maize cultivation on the communities of the non-target AMF in the roots and soils.

The dominance of *Glomus* in the AMF communities of most of the samples in our study was also common in other ecosystems [48, 51, 52], which further indicated that Bt maize did not have a significant influence on the composition of the AMF community. The rare *Rhizophagus* was only observed in the rhizospheric soil of 5422Bt1 and the roots of 5422 (Fig 4). This might result from the soils used for cultivation or the host preferences of AMF [29]. Other factors, such as the physical and chemical features of the soil and agricultural management, can also influence the structure and composition of the AMF community in agricultural systems [40, 53, 54].

The phylogenetic tree of the AMF genera did not show obvious clusters according to the maize cultivars, indicating that the introduction of the Bt gene into maize did not significantly affect the AMF phylotypes. However, the phylogenetic tree contained clades that corresponded to the sample types (roots, bulk soils or rhizospheric soils). This suggested that the sample types might have a greater influence on the AMF community phylotypes than the maize cultivars, and the AMF communities were spatially heterogeneous in the soils. This idea was supported by the results of previous studies, in which the AMF spores and hyphae were distributed in a loosely-clustered or loose dot manner in the soils [54], as well as the phenological characteristics of the germ-tube (its germination and cessation of growth in the soils and roots), and the infective unit of the AMF hyphae [22].

A few studies of the long-term effects of GM crops on non-target microorganisms have been reported. Barriuso et al. [55] documented that there were no differences between Bt (DKC6451YG [MON810]) and non-Bt maize (DKC6450) in the diversity and structure of rhizobacterial communities over a period of four years (one season per year, a total of four crop seasons within four years), as determined by high throughput DNA pyro-sequencing. Hannula et al. [56] conducted a 3-year study of potatoes with different GM traits (one season per year, a total of three crop seasons within three years). They found that the plant growth stage, season, and field site affected the fungal communities, including AMF communities, whereas the cultivar and GM-trait (amylopectin-accumulating) had only minor effects. Our study also revealed that continuously cultivated Bt maize had minor effects on AMF colonisation and community structure.

Assessing the effects of GM plants on non-target organisms is a long-term process. Our future studies will focus on examining the AMF community structure in the roots and soils of Bt maize for additional consecutive crop seasons in order to evaluate the long-term effects and potential ecological risks of Bt maize cultivation on non-target AMF microbes.

## Conclusions

The AMF colonisation was significantly higher in roots of the two Bt maize cultivars (5422Bt1 and 5422CBCL) than in the non-Bt isoline 5422 planted in the fifth season of continuous Bt maize cultivation. No significant differences were observed in the diversity of the AMF community in the roots, rhizospheric soils and bulk soils from the Bt and non-Bt maize cultivars, as demonstrated by the T-RFLP analysis. The AMF *Glomus* was dominant in most of the samples, as detected by DNA sequencing of the 16S rDNA fragment. This study suggested that the Cry1Ab protein has minor effects on the AMF communities after five seasons of continuous Bt maize cultivation.

## Supporting Information

S1 Fig Nonmetric multidimensional scaling (NMDS) ordination plots of the AM fungal communities in the roots, rhizospheric soils, and bulk soils harvested at the fifth season, which are based on the TRFs for each enzyme. The filled square represents 5422, the circle represents 5422Bt1, and the triangle represents 5422CBCL. The key indicated the formula: sample type-restriction enzyme. A = bulk soils; B = rhizospheric soil; C = roots; 1 = *Mbo*I; and 2 = *Taq*I.
(DOCX)

S1 Table Primers used in these experiments. (XLSX)

S2 Table Closest relatives in maize associated with the Bt and non-Bt lines after five seasons of continuous cultivation. (XLS)

S1 Text Highlights of the paper entitled “The Cry1Ab Protein Has Minor Effects on the Arbuscular Mycorrhizal Fungal Communities after Five Seasons of Continuous Bt Maize Cultivation.” (DOC)
