# Supplementary material for: The Cry1Ab Protein Has Minor Effects on the Arbuscular Mycorrhizal Fungal Communities after Five Seasons of Continuous Bt Maize Cultivation
Source: PLoS One. 2015 Dec 30;10(12):e0146041. doi: 10.1371/journal.pone.0146041 (PMC4696834; doi:10.1371/journal.pone.0146041)
Supplement: S1 Fig — The filled square represents 5422, the circle represents 5422Bt1, and the triangle represents 5422CBCL. The key indicated the formula: sample type-restriction enzyme. A = bulk soils; B = rhizospheric soil; C = roots; 1 = MboI; and 2 = TaqI. (DOCX) [file pone.0146041.s001.docx]

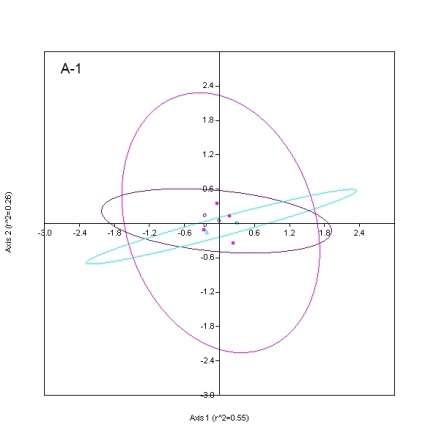

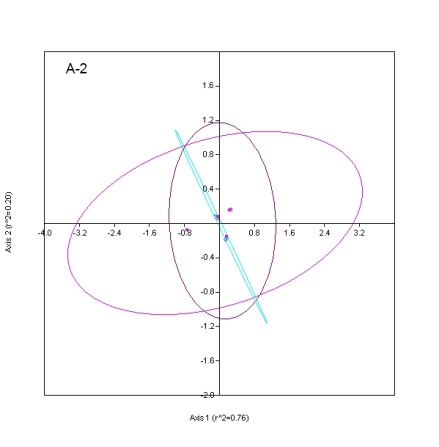

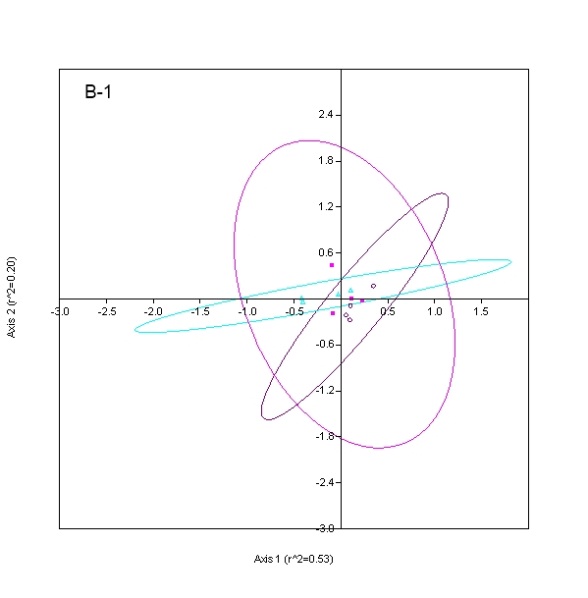

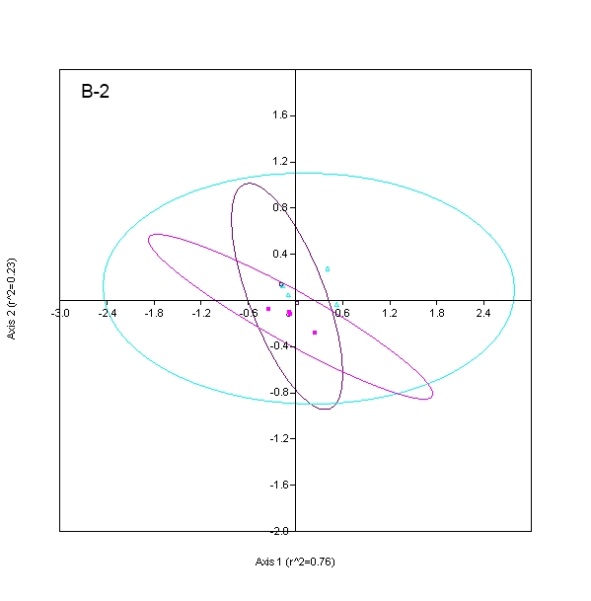


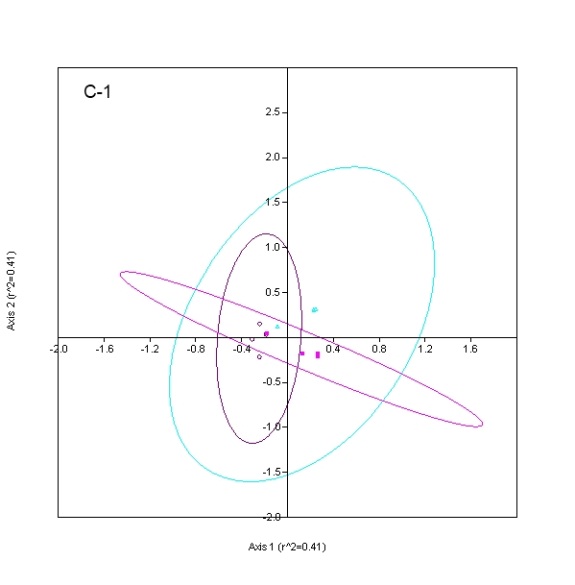

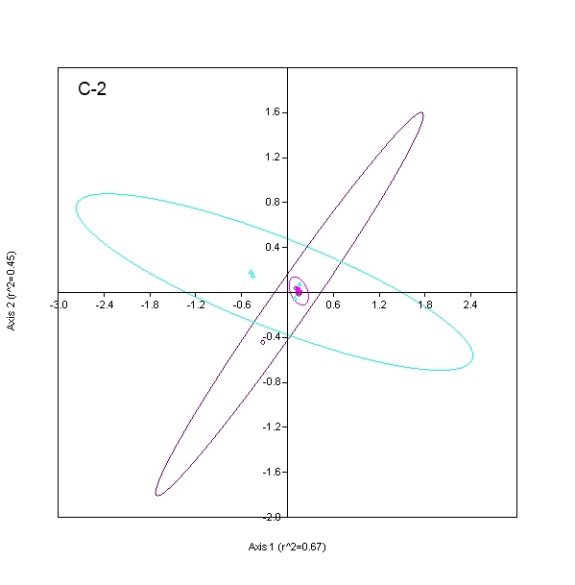


**S1 Fig. Nonmetric multidimensional scaling (NMDS) ordination plots of the AM fungal communities in the roots, rhizospheric soils, and bulk soils harvested at the fifth season, which are based on the TRFs for each enzyme.** The filled square represents 5422, the circle represents 5422Bt1, and the triangle represents 5422CBCL. The key indicated the formula: sample type-restriction enzyme. A = bulk soils; B = rhizospheric soil; C = roots; 1 = *Mbo*I; and 2 = *Taq*I. (DOCX)
