# Supplementary material for: The Cry1Ab Protein Has Minor Effects on the Arbuscular Mycorrhizal Fungal Communities after Five Seasons of Continuous Bt Maize Cultivation
Source: PLoS One. 2015 Dec 30;10(12):e0146041. doi: 10.1371/journal.pone.0146041 (PMC4696834; doi:10.1371/journal.pone.0146041)
Supplement: S1 Text — (DOC) [file pone.0146041.s004.doc]

**Text S1. Highlights of the paper entitled “The Cry1Ab Protein Has Minor Effects on the Arbuscular Mycorrhizal Fungal Communities after Five Seasons of Continuous Bt Maize Cultivation”**

- Two Bt maize lines and a non-Bt isoline were continuously cultivated for 5 seasons.
- AMF colonization was higher in the two Bt maize lines than in the non-Bt isoline.
- AMF community diversity and structure did not differ between Bt and non-Bt plants.
- Continuous Bt maize cultivation had minor effect on AMF colonization.
